# Dog ownership practices and responsibilities for children’s health in terms of rabies control and prevention in rural communities in Tanzania

**DOI:** 10.1371/journal.pntd.0009220

**Published:** 2021-03-10

**Authors:** Lwitiko Sikana, Tiziana Lembo, Katie Hampson, Kennedy Lushasi, Sally Mtenga, Maganga Sambo, Daniel Wight, Jane Coutts, Katharina Kreppel

**Affiliations:** 1 Environmental Health and Ecological Sciences, Ifakara Health Institute, Ifakara, Tanzania; 2 Global Health and Biomedical Sciences, School of Life Sciences and Bio-Engineering, Nelson Mandela African Institution of Science and Technology, Arusha, Tanzania; 3 Boyd Orr Centre for Population and Ecosystem Health, Institute of Biodiversity, Animal Health & Comparative Medicine, University of Glasgow, Glasgow, United Kingdom; 4 Medical Research Council/Chief Scientist Office (MRC/CSO), Social and Public Health Sciences Unit, University of Glasgow, Glasgow, United Kingdom; University of Surrey, UNITED KINGDOM

## Abstract

Interventions tackling zoonoses require an understanding of healthcare patterns related to both human and animal hosts. The control of dog-mediated rabies is a good example. Despite the availability of effective control measures, 59,000 people die of rabies every year worldwide. In Tanzania, children are most at risk, contributing ~40% of deaths. Mass dog vaccination can break the transmission cycle, but reaching the recommended 70% coverage is challenging where vaccination depends on willingness to vaccinate dogs. Awareness campaigns in communities often target children, but do not consider other key individuals in the prevention chain. Understanding factors related to dog ownership and household-level responsibility for dog vaccination and child health is critical to the design of vaccination strategies. We investigated who makes household decisions about dogs and on health care for children in rural Tanzania. In the Kilosa district, in-depth interviews with 10 key informants were conducted to inform analysis of data from a household survey of 799 households and a survey on Knowledge Attitudes and Practices of 417 households. The in-depth interviews were analysed using framework analysis. Descriptive analysis showed responsibilities for household decisions on dogs’ and children’s health. Multivariate analysis determined factors associated with the probability of dogs being owned and the number of dogs owned, as well as factors associated with the responsibility for child health. Dog ownership varied considerably between villages and even households. The number of dogs per household was associated with the size of a household and the presence of livestock. Children are not directly involved in the decision to vaccinate a dog, which is largely made by the father, while responsibility for seeking health care if a child is bitten lies with the mother. These novel results are relevant for the design and implementation of rabies interventions. Specifically, awareness campaigns should focus on decision-makers in households to improve rabies prevention practices and on the understanding of processes critical to the control of zoonoses more broadly.

## Author summary

The design of interventions tackling diseases transmitted between animals and humans requires an understanding of healthcare patterns related to both human and animal hosts. The control of dog-mediated rabies is a good example of this need. Rabies is still a major problem in Tanzania, where children are most at risk, and more than 40% of deaths are in children under 15 years old. While the vaccination of dogs against rabies is an effective method for controlling the disease, dog vaccination coverage is too low and depends on the willingness of dog owners to vaccinate. If a person is bitten by a rabid dog, the only way to prevent death is to receive post-exposure prophylaxis (PEP) in time. Hence, understanding factors related to dog ownership and responsibility for dog vaccination and child health is critical to the design of vaccination strategies. This study found that it is important to understand the role of these processes critical to the control of zoonoses, so that disease prevention campaigns can focus on a range of decision-makers, particularly those in households.

## Introduction

Zoonotic pathogens shared between human and animal populations are widespread in the developing world. The multi-host nature of these pathogens has a dual impact on human and animal health in terms of illness, death and the high costs of care [1]. In many cases a single animal host is responsible for most of the transmission to humans, which offers opportunities for integrated, yet focused, action towards control and eventual elimination of the threat, using methods such as comprehensive vaccination of the animal host population. In the meantime, however, direct protection of individuals exposed to infected animals is also necessary. Therefore, an understanding of healthcare patterns related to both human and animal hosts is critical for public-health interventions tackling zoonoses.

Rabies exemplifies these issues well. It is a fatal zoonosis caused by a *Lyssavirus* and can affect all mammals, including humans [2]. The disease presents a significant public health risk globally, with an estimated 59,000 deaths occurring worldwide every year [3,4]. The most affected continents are Africa and Asia [1–3]. In Tanzania, for example, the disease is responsible for causing approximately 2,000 deaths annually, of which 40% are children below the age of 15 [5], and most deaths reported are from the poorest households in rural areas [6–8]. The main source of human rabies is a bite from an infected domestic dog (*Canis familiaris*) [9].

Rabies is entirely preventable through vaccination of dogs and immediate administration of wound management and post-exposure prophylaxis (PEP), comprising administration of rabies immunoglobulin and rabies vaccine to humans exposed to suspected rabid bites. In particular, mass dog vaccination (MDV) is an effective strategy in preventing rabies in both species, but, as with other immunisation programmes, sustained participation in successive vaccination campaigns is required to achieve herd immunity. At least 70% of susceptible dogs must be vaccinated to break the rabies transmission cycle [10–13]. Rabies-control initiatives for vaccinating dogs in endemic areas are typically carried out by the private sector and international bodies such as the World Health Organization (WHO), Food and Agriculture Organization (FAO), World Organisation for Animal Health (OIE) and Global Alliance for Rabies Control (GARC) [9]. Unfortunately, these vaccination campaigns struggle to reach or sustain the 70% vaccination coverage needed to control dog-mediated rabies successfully [12,14]. To overcome these challenges, in many countries national action plans are being developed in line with global initiatives for rabies elimination by 2030 [9]. For instance, in Tanzania, a national rabies elimination strategy approved in December 2019 (Chinyuka, H., personal communication, December 20^th^ 2019) will ultimately involve a nationwide vaccination campaign which will require dog owners in the country to vaccinate their dogs. However, until then, dog vaccination is not compulsory and is not always free of charge.

Previous studies in Africa have highlighted that vaccination efforts are influenced by local dog-ownership practices [15]. The few studies that have addressed responsibilities surrounding dog ownership in Africa have reported differences across locations, particularly between rural and urban settings [16,17]. In Tanzania the agricultural sector contributes 56% of the country’s domestic income and the livelihoods of approximately 3.6 million people depend on it [18]. This includes communities that fully or partly depend on livestock. As in most of sub-Saharan Africa, dogs are widely used for herding, and protecting families and livestock against wild animals and thieves [19]. Differences in livelihood patterns between a range of settings (e.g. town, farmland and pastoral) have been demonstrated to influence human-dog relationships and the spatial distribution of dogs [17]. A more detailed understanding of practices associated with dog ownership will be an important step towards effective design and implementation of MDV. Of particular interest is the value of dogs to households and how decisions are made at the household level about whether to vaccinate them.

Another critical area requiring investigation relates to household decision making around children’s health, and the role children could play in preventing rabies in rural Africa. Children aged 6–12 years are the primary victims of rabies [5,20], and are also most likely to take care of dogs and bring them for vaccination [21]. Fortunately, the development of rabies in individuals bitten by suspect rabid dogs can be prevented through immediate administration of post-exposure prophylaxis (PEP). However, even if children understand the need to report bites and respond promptly to being bitten, there is insufficient information on their ability to influence the decision to do so.

To shed light on this, data were extracted from a household survey and a Knowledge, Attitudes and Practices (KAP) survey. However, this process was preceded by an initial stage involving key-informant interviews (KII). These were intended to provide pointers to the most relevant questions to investigate using the survey data, by highlighting important issues from the point of view of local health and veterinary staff with experience of working in the area, as well as village decision-makers. In terms of this paper, the most significant questions to emerge from this process focused on who makes household decisions about dogs and who seeks health care for children. These questions were broken down into the following areas for analysis: who keeps dogs; who is responsible for them; what influences the number kept; why dogs are valued; and who is responsible for the associated risks to children’s health. The conclusions from the analysis of these data will provide a basis for future research and for targeting vaccination interventions more strategically.

## Materials and methods

### Ethics statement

Ethical approval was obtained through the Ethical Review Board of the Ifakara Health Institute- (IHI/IRB/No.23-2014) and the National Institution of Medical Research (NIMR), Tanzania (NIMR/HQ/R.8a/Vol. IX/2200). Additionally, permission for data collection at the district, village and sub-village level was obtained from the respective authorities. Participation in this study was voluntary. Participants were adults, and before the questionnaire was administered they were given information sheets explaining the purpose and procedures of their study as well as their roles. For qualitative data collection, verbal consent was acquired from each participant. All information from this study remains confidential, and qualitative data responses were coded to prevent identification of respondents.

### Study setting

**Fig 1:**
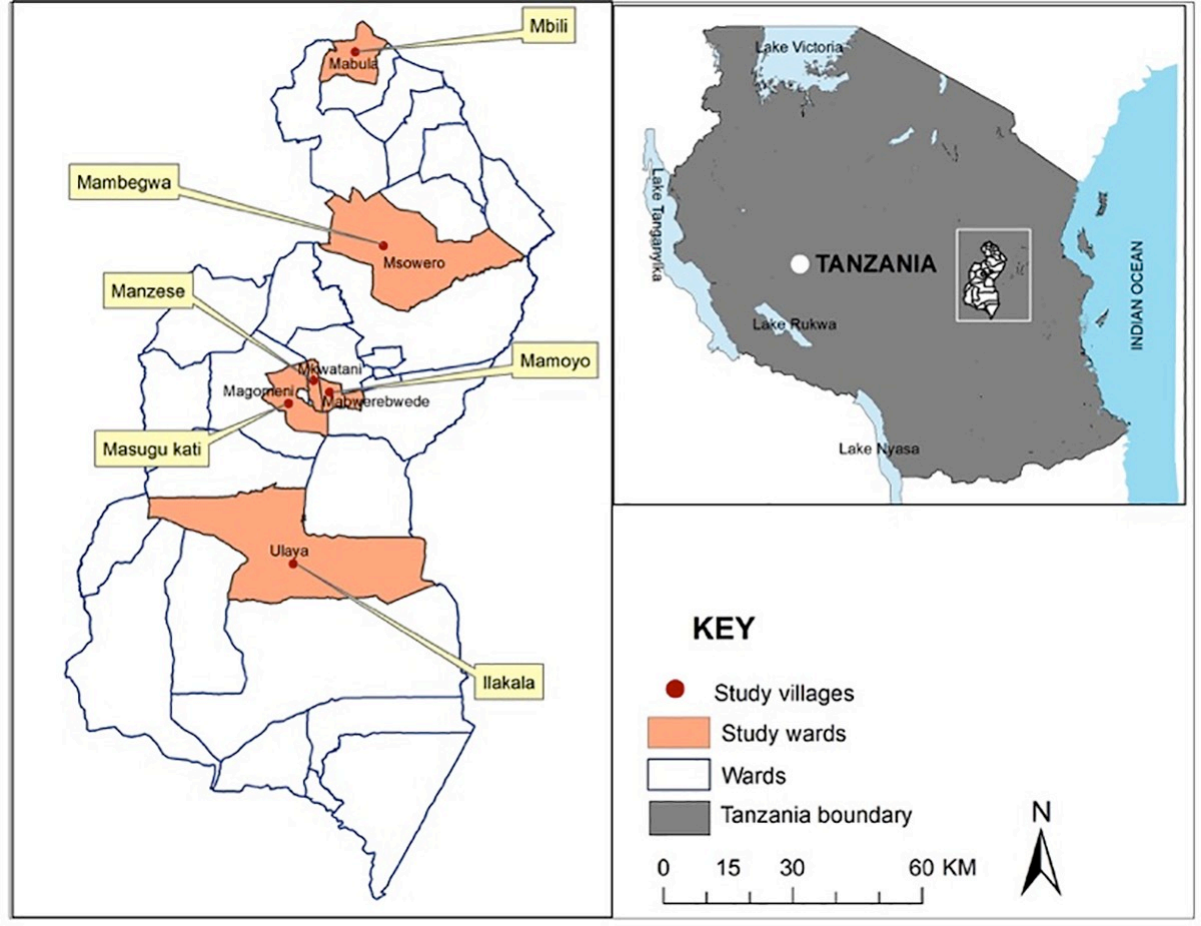
Map showing the location of the study area in Kilosa district, Tanzania, including the study wards and villages.

The study was conducted in the Kilosa District (6.8343° S, 36.9917° E), in the western part of Morogoro Region in Tanzania (Fig 1). Kilosa was chosen because of previous study findings of low awareness of rabies in the area [22]. The study area covers about 14,400 km^2^, with an estimated population of 438,175 and 105,635 households [22,23]. In Kilosa, most villagers have a pastoral background and keep livestock, mainly cattle, but also goats and sheep, while some are subsistence-agriculture farmers. The District consists of 38 wards with 3,570 to 29,361 people per ward [24]. The area experiences a long dry season from June to October, and two rainy seasons (December to January and March to May) with an average of 976mm of rain per year (Fig 1). Heavy rains regularly make transport to major health centres difficult and can influence the timing of dog vaccination campaigns.

### Data collection

A mixed-methods approach was used to collect quantitative and qualitative data. First, field interviewers from the Ifakara Health Institute collected qualitative data through 10 audio-recorded semi-structured KII. The KII included questions on the examination and treatment procedures followed by staff at public-sector livestock/veterinary offices and local dispensaries, in the event of presentation of a suspected rabies case, along with their experiences involving children’s health and mass dog vaccination (S1A Text). The target group for the interviews with the health sector (n = 4) was from Ilakala, Manzese, Masugu Kati and Mamoyo villages. The interviews with veterinary authorities (n = 2) involved the Kilosa district authority and Mamoyo villages. Finally, village-level participants (n = 4) were from Mamoyo, Masugu Kati, Manzese and Mbili villages. The interviews took place between November 2014 and February 2015 under the supervision of the lead author. An inductive analysis of these data informed the specific relationships to explore in analysing the household survey data. Where the general focus of the investigation had been informed by recommendations in recent literature, the KII process went some way to ensuring that the issues taken forward for quantitative analysis were relevant from the point of view of those working on the ground.

The same five trained field-interviewers from the Ifakara Health Institute also collected data for a household survey (HHS, March-April 2015) and a KAP survey (KAP, January-February 2016). A cross-sectional study design was used for structured HHS and KAP surveys. Questionnaires in hard copy were administered and filled out by the team of interviewers. Participants’ demographic data were anonymised. Six wards were chosen randomly (Fig 1). From each ward, one village was then selected at random: Mamoyo, Ilakala, Masugu Kati, Mbili, Mambegwa and Manzese. Of these, 5% of the village households (n = 1216) were randomly chosen for questionnaire administration for both the HHS and the KAP survey. The household questionnaire included questions on demographics, sources of income, livestock ownership, dog ownership, responsibilities for dogs at the household level, responsibilities for children and child health-care patterns in terms of dog-bites (S1B and S1C Text). Questions specifically intended for the KAP survey involved household members’ understanding of rabies prevention and how they put this into practice. The results were intended as a baseline for measuring any changes at a later date. Questionnaires were administered to the head of household (18+ years) or another adult resident in the same household on behalf of the head.

### Analyses

#### Quantitative data

Quantitative data were entered using Epi-Info version 5.2 (Centers for Disease Control and Prevention, Atlanta, Ga, USA) and transcribed into “Microsoft Excel” version 16.23 (www.microsoft.com). Statistical analyses were performed using R version 3.6.1 [25]. Data from both surveys were merged where appropriate using the unique identifier from each household. Responses to questions asked in both surveys given by the same household were cross referenced. Descriptive analysis was carried out to obtain the general characteristics of the study population (Table 1). Characteristics of the data on responsibility for dog ownership and for the health of children in the household were summarised and plotted. To understand which individuals are largely responsible for household decisions about dogs and child health, and who takes dogs for vaccination, only data from households with dogs were included.

**Table 1:** Characteristics of the respondents and of their households as collected by the Household Survey (HHS) and Knowledge Attitudes and Practices Survey (KAP) respondents and their households.

Variables KAP and HHS	Frequency	Percentage (%) of the total
**Gender of respondents (n = 1216)** Male Female		
	427 789	35.1 64.9
**Age groups of respondents (n = 1216)** Range Median 18–35 years 36–50 years > 50 years		
	18–99 35 644 339 233	NA NA 52.9 27.9 19.2
**Household size (n = 1216)** <6 >6		
	878 338	72.2 27.8
**Households owning dogs by village (n = 325)** Ilakala Mambegwa Mamoyo Manzese Masugu Mbili		
	73 77 6 57 18 94	22.5 23.7 1.8 17.5 6.9 28
**Number of households (n = 798) owning each type of livestock** Dog Cattle Goat Sheep Pig Chicken Cat Other livestock*		
	126 63 84 10 26 692 129 89	15.7 7.9 10.5 1.2 3.2 86.7 16.2 11.1
**Variable HHS only**	**Frequency**	**Percentage (%)of the total**
**Religion (n = 799)** Christian Muslim Traditional beliefs NA		
	491 297 2 9	61.4 37.2 0.25 1.13

* E.g. Pigeons, ducks and rabbits.

Multivariate analyses were used to test for associations between variables (p-value ≤0.05). For this, generalised linear models (GLMs) were fitted.For factors associated with dog ownership, data were combined from the HHS and KAP, resulting in a total of 1216 unique households, to determine predictors of dog ownership and factors affecting the number of dogs owned. For models containing the response variables “dog ownership” and “number of dogs owned”, the explanatory variables “village”, “cattle”, “pig”, “sheep and goats”, “other livestock”, “chickens” and “household size” were included. The data for the response variable “dog ownership” were modelled using a binomial distribution, resulting in odds ratios. For the response variable “number of dogs owned”, a Poisson distribution was used. A glm with binomial distribution was used to assess the relationship between the family member responsible for children’s health if they are bitten, and village and household size.

The final models were selected based on the rule of parsimony, and by using a likelihood ratio test (LRT) with a significant p-value of ≤ 0.05, as well as visual inspection of the residuals for the fitted-model [26,27].

#### Qualitative data from interviews

Interviews were recorded using a digital tape recorder and notes were taken throughout the conversations. They were analysed as part of a broader project, using framework analysis [28], and were made available for use in the current paper. All interviews were conducted in Swahili, and conversations were transcribed and translated into English. Data were entered using Microsoft Office 2013, and themes were developed according to the relevance of the study topics. The findings from the qualitative data were used to inform the quantitative analyses of the household survey data, and in some cases to shed further light on the results of quantitative analyses.

## Results

Qualitative data from the key-informant interviews highlighted a number of relevant points to inform further exploration using the quantitative data:

Veterinary officers mentioned that “pupils up to standard five are very active on bringing dog(s) for vaccination”. (ID# A 001).

- There was nevertheless no information from the interviews on the child’s role in deciding to bring the dog for vaccination.
- Veterinary officers noted that the children were given information about vaccination campaigns in terms of cost and logistics. However, it was unclear whether the officer in question knew what happened to this information when the child went home, or how it was taken forward in the household, and this was not followed up in the interview. It therefore posed a question for further, quantitative, investigation in terms of who makes decisions in households.
- Mothers were cited as those more likely to accompany a child to a health facility: *“Often a person who escorts a child to a health facility is a mother”* (ID# C 002). Once again, however, it was unclear whether the mother was actually the person who made the decision, and this needed to be followed up quantitatively.
- A respondent at Mabwerebwere Dispensary (I.1) had not recently heard of any rabies cases in the area, but thought most cases happened in children. He thought it possible there were cases in children in the area which were not reported. Again, this pointed to the need for further, quantitative, investigation of decision-making processes in households, and attitudes towards reporting bite cases.
- Respondents in the health sector indicated that a child with a dog bite could be brought into the health facility by their father, their mother or a guardian. A respondent at Berega Missionary Hospital (I.11) suggested that, although most came with their mother, children 3–6 years might come with their father. *“Children are generally brought by their mother*, *but if they are 3–6 years*, *they might come with the father*. *Instructions are normally given to the parent”*. (ID# C 003). However, the interviews did not indicate the specific relationship between who brought a child in for vaccination and the person in the household who made the decision to do so. This needed to be explored further in the quantitative data.

These results were extracted as the most relevant to pursue in terms of quantitative analysis, alongside background analysis of trends in dog ownership.

### Characteristics of the respondents and variables

Of the 1216 individual households from the HHS and KAP surveys in the six villages, 65% owned livestock and 26% reported owning dogs. Among the households owning livestock, 16% reported also owning dogs. Of the 798 households owning livestock, the majority owned chickens (86.7%) followed by cats (16.2%) and dogs (15.7%). Of the 126 households owning dogs, 42% also owned cattle, goats or sheep–animals traditionally herded. There were significant differences between villages in terms of the number of dogs owned (Table 1).

### Factors associated with dog ownership

Table 2 shows that the village where the respondent lived was significantly associated with the likelihood of dog ownership.

**Table 2:** Predictors associated with the probability of dog ownership in six villages in rural Tanzania, with 95% CI and p-values.

Variable	OR	CI 2.5%	CI 97.5%	p-value
Ilakala village	1			
Mambegwa village	0.56	0.31	1.03	0.0621
Mamoyo village	0.12	0.04	0.57	0.009
Manzese village	0.84	0.47	1.52	0.554
Masugu village	3.94	1.46	9.96	0.005
Mbili village	1.22	0.64	2.3	0.546
Household size	1.31	1.21	1.44	<0.001
Cattle	3.61	1.92	6.67	<0. 001
Pig	2.84	1.09	6.88	0.024
Other livestock	2.44	1.3	4.39	0.004

While living in Mambegwa or Mamoyo village was associated with a lower probability of owning a dog (OR = 0.56, p-value = 0.062 and OR = 0.12, p-value = 0.009 respectively), living in Masugu village was associated with a significantly higher probability of owning dogs (OR = 3.94, p-value = 0.005). Dog ownership was positively associated with household size (OR = 1.31, p-value <0.001) and livestock ownership (Tables 2 and 3). Having cattle greatly increased the odds of owning a dog (OR = 3.61, p<0.001), followed by keeping pigs (OR = 2.84, p = 0.024) and other livestock (OR = 2.44, p = 0.004; Tables 2 and 3). Owning goats and sheep was not associated with dog ownership.

**Table 3:** Likelihood ratio test χ^2^ values of all significant variables in the final glm on the probability of owning dogs.

Variable	χ^2^	p-value
Village	27.4	<0.001
Household size	38	<0.001
Cattle	15.25	<0.001
Pig	4.56	0.032
Other livestock	7.49	0.006

### Factors associated with the number of dogs owned

**Fig 2:**
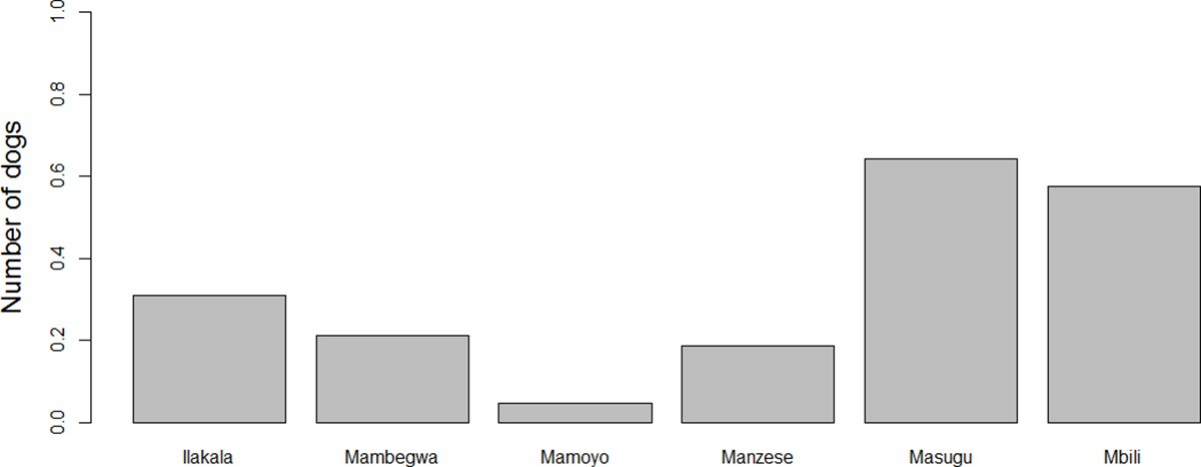
Average number of dogs per household in each of the six villages within the study district, Kilosa, Tanzania.

The number of dogs per household differed significantly by village (Fig 2, Tables 4 and 5). Living in Mambegwa, Mamoyo or Manzese was negatively associated with the total number of dogs owned compared to living in Ilakala. Living in Masugu or Mbili was not significantly different to Ilakala with regard to the number of dogs owned. Household size was positively associated with the number of dogs kept (Tables 4 and 5). There was also a positive association between the number of dogs owned and sheep, cattle and pig ownership (Tables 4 and 5). Owning goats and other livestock was not significantly associated with the number of dogs owned per household.

**Table 4:** Predictors associated with the number of dogs owned per household in six villages in rural Tanzania, with 95% CI and p-values.

Variable	Mean estimate	CI 2.5%	CI 97.5%	p-value
Ilakala village	ref	NA	NA	NA
Mambegwa village	-0.16	-0.32	-0.001	0.0486
Mamoyo village	-0.26	-0.472	-0.05	0.0145
Manzese village	-0.19	-0.36	-0.02	0.0235
Masugu village	0.34	-0.03	0.73	0.0710
Mbili village	-0.02	-0.22	0.18	0.8327
Household size	0.07	0.05	0.1	<0.0001
Sheep	0.74	0.16	1.33	0.0119
Cattle	0.71	0.45	0.973	<0.0001
Pig	0.63	0.27	1.00	0.0007

**Table 5:** Likelihood ratio test χ^2^ values of all significant variables in the final glm associated with the number of dogs owned.

Variable	χ^2^	p-value
Village	16.79	0.005
Household size	33.47	<0.001
Sheep	6.37	0.01
Cattle	28.4	<0.001
Pig	11.57	<0.001

In interviews in Mamoyo village, a respondent suggested reasons why fewer people owned dogs there:

*“In our village*, *there are fewer dogs*, *because most of the people are Muslims*, *they are not keeping dogs*.” *(ID# C 001)*

Looking at the qualitative interviews from the key informant interviews, only one Muslim household in Mamoyo village kept a dog. We also noted that few, if any, of the Muslim households kept livestock, a factor associated with dog ownership. However, this relationship was not taken further in the quantitative analysis, as data were insufficient to produce a meaningful result, but the issue merits further attention.

### Responsibilities for dog vaccination

Of the 417 households visited during the KAP survey, only 66 owned dogs and the total number of dogs were 162, with 83% reported to be vaccinated against rabies.

**Fig 3:**
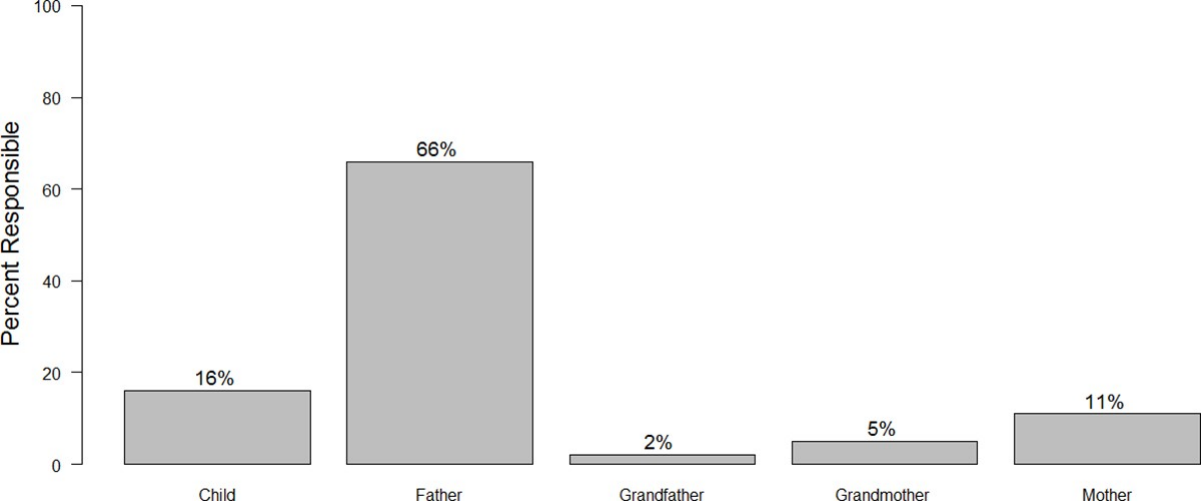
Responsibility of household members for dog vaccination decision.

In response to other survey questions, 53 heads of households gave information on responsibilities for vaccinating dogs and for decisions regarding children’s health at the household level. In a majority of households with dogs, the respondent reported that the father decides whether to vaccinate the animal (66%), followed by 16% of households where a child decides and just 11% where the mother is the decision maker (Fig 3).

Children, on the other hand, are likely to take dogs for vaccination, confirming the comments noted above in the key-informant interviews. (ID# A001)

**Fig 4:**
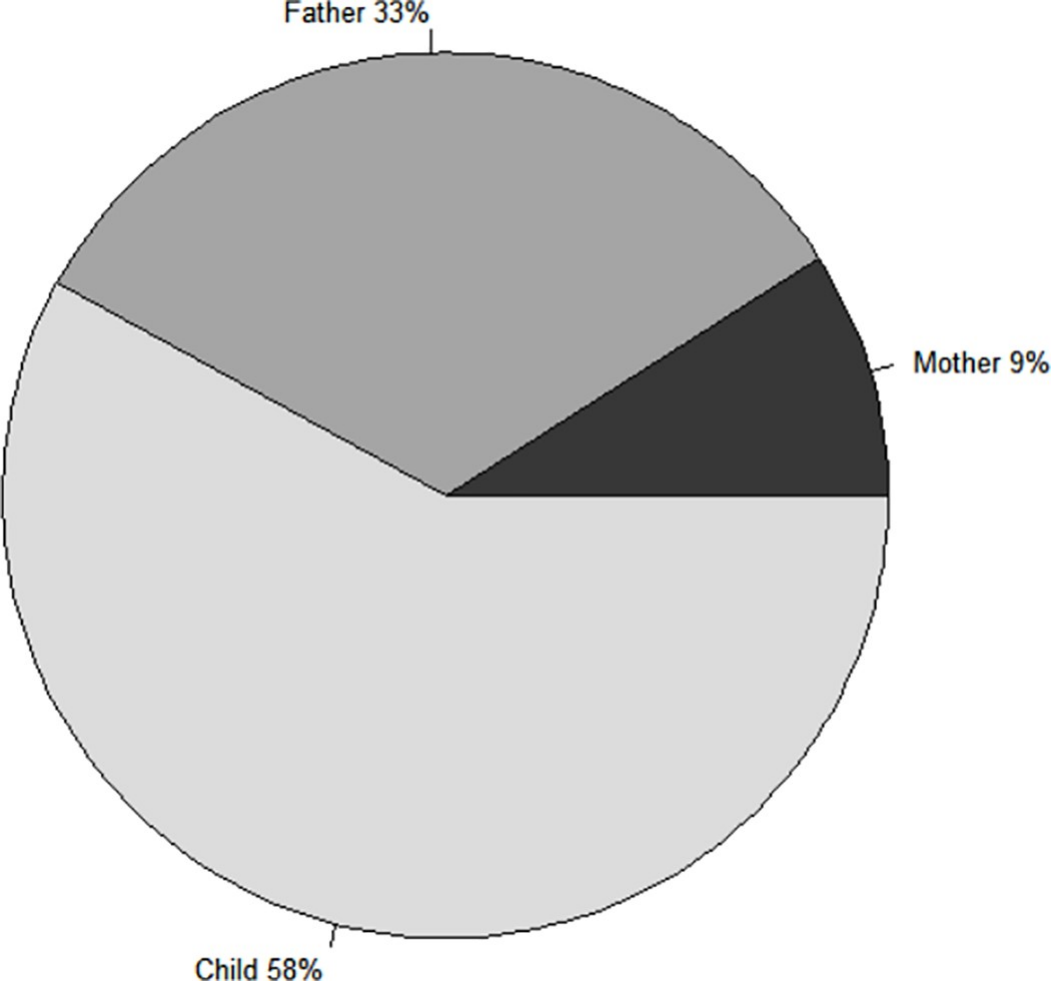
Household member who takes dogs to the vaccination point.

In the 64 dog-owning households which answered the question: “Who takes dogs for vaccination?”, 58% said that this was undertaken by children, 33% said the father and only 9% of households reported that it was the mother (Fig 4).

In the KIIs, the veterinary authorities had been asked about their procedures in terms of organising mass dog vaccination. They noted that they informed people visiting the veterinary office on Saturdays about dog vaccination dates. There was no indication that this involved any exchange of information on difficulties households might experience in attending, and seems to have been a simple notification of dates and times.

*“So*, *we advertise it to all who come here every Saturday … If there is a cost of vaccinating*, *people are informed directly about the cost and if it is a free service people will be informed*. *That depends on the owner of the vaccine*, *if it is a council or privately owned*. *We have never supplied or bought vaccine*. *That is why we normally conduct the campaign on cost*.” *(ID# A 001)*

Scheduled village meetings are also a conduit for disseminating information about vaccination campaigns, but the extent of the information is unclear, as is the issue of how it is passed on to people beyond those attending the meeting.

**“***Normal meetings are held every three months*, *for village leaders*. *Health messages can be incorporated after the normal business … (ID# A 002)*

### Responsibilities for children’s health if they are bitten by a dog

**Fig 5:**
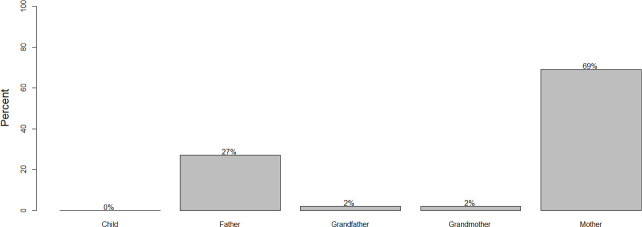
Household member responsible for decisions on seeking health care for children bitten by dogs.

In 69% of households the mother decides on the course of action when a child is bitten by a dog (Fig 5). However, while in general the mother seems to decide, increasing household size increases the probability of the father making decisions on treatment and PEP of children (coef = 0.47, p-value = 0.004, χ^2^ = 11.22, p-value = 0.0008). Incidences of dog bites in children mostly take place during the morning when they are on the way to school, or in the afternoon when they are going home. Mothers were generally at home, so they tended to be the first point of contact for a child when an accident happened.

## Discussion

This study investigated dog ownership and care patterns in the context of mass dog rabies vaccination, as well as household responsibilities for children’s health, in order to inform rabies prevention interventions.

The results show that: (1) larger households with livestock including cattle are more likely to own dogs and a greater number of dogs compared to households owning other livestock such as chickens, cats or goats; (2) a child is likely to take a dog to the vaccination point, but (3) the decision on whether a household dog will be vaccinated against rabies usually lies with the father; (4) a child’s mother typically decides on the course of action if the child is bitten by a suspected rabid animal.

These findings are in line with those of other studies which show engaging in farming activities and keeping a greater number of livestock are positively correlated with higher dog numbers in rural areas [16,29]. This may be because of a greater need to guard livestock, and also the fact that more food is available to feed dogs. In Bali, Indonesia, it was reported that dogs are used as security at home and in the plantations, as well as for companionship [30]. Related heterogeneities in dog ownership were observed in Kenya [31]. Both in rural and urban Tanzania, dogs are kept as a safeguard against thieves [16]. We show that the larger the household, the greater the number of dogs kept, similar to a study in Ethiopia [15]. The number of dogs owned may also relate to religion. For instance, Muslim communities were likely to own fewer dogs as these animals are considered ritually unclean. However, insufficient Muslim households answered relevant questions in the current study to validate this result.

The findings of the present study show that responsibility for dog vaccination is shared between family members, but children are not directly involved in the decision-making processes. Children tend to take dogs to vaccination points, particularly children aged between 5 and 15, but decisions on dog vaccination are mainly made by the father. It would be interesting to see if the father’s decision varies depending on the cost of the vaccination. In Chad, Durr et al. [32] found more dogs were vaccinated when rabies vaccination was free. Meanwhile, it is generally the mother who decides whether to access health services if a child is bitten. This also applies to other infectious diseases, and it is mainly the mother who takes a child to health services [33]. This agrees with a study which showed that responsibility for dog ownership lies with household members over 18 years of age [29]. On the other hand, children are the group most at risk from dog bites [31], and male children seem to be bitten more often than female children [34,35]. Many interventions, therefore, target children, often through schools, generally involving awareness raising [36,37]. It is also often suggested that increasing knowledge about rabies among children could have a strong impact on preventative measures such as vaccination [38]. The findings of the present study suggest, however, that vaccination and awareness campaigns may need to include more directly those who make household decisions about children’s and animals’ health, rather than relying on children only as a conduit for information. Children are unlikely to have any reliable or widespread form of influence on how the information is taken forward in the household, especially given that several studies on sexual and reproductive health issues found that children have low status within rural families and limited child-initiated conversation with their parents [39–41]. However, more research into the dynamics of communication between children and their parents is needed to ascertain this when it comes to other diseases.

A community survey carried out in southern Tanzania looked at willingness to pay for dog vaccination against rabies, but the suggested amount was insufficient to cover actual vaccination costs, and findings suggested that means of collecting contributions could be a challenge [42]. Another study revealed that few dog owners were asked about their willingness to participate in mass dog vaccination campaigns [17]. In addition to understanding willingness to vaccinate dogs, socio-economic factors should be taken into account which influence whether households are in a position to do so [7,17]. Highlighting socio-economic obstacles to compliance, and developing mechanisms to address them, will be essential if disease control and prevention in general is to be sustainable in communities.

The results of the current study show that, while messages delivered to children may be valuable in terms of more general rabies prevention information, such as avoiding dog bites, there is no indication that more specific information about vaccination or seeking PEP is reaching, or influencing those who specifically make the decisions about it. This particularly includes those who are not currently in favour of vaccinating dogs, or those with difficulties seeking PEP due to limited resources or time.

Qualitative data showed that community meetings or gatherings are used to communicate messages about rabies vaccination, and veterinary officers noted that these are essential places for sharing information which could influence people to bring their animals in for vaccination against rabies and other diseases. However, the extent to which these general forms of communication are directly reaching household decision-makers remains unclear, particularly anyone who has doubts about vaccination. Furthermore, the information appears to be one-way, and there is no indication whether mechanisms exist for investigating or recording obstacles faced by households in terms of agreeing to vaccination, even if they are aware of the importance of it. In other words, the focus seems to be on awareness, rather than assessing feasibility and attitude. A study conducted in South-eastern Tanzania suggested that, in planning MDV interventions, it is important to understand the dynamics involved in people’s willingness to vaccinate household dogs [17]. Understanding these factors, and working towards formal mechanisms for this type of consultation, will help target rabies control and prevention more effectively in communities.

This points to three areas for consideration. First, there should be further research on the dynamics which specifically influence a household decision to vaccinate a dog. Data from other studies point to the fact that many influential factors are likely to be socio-economic [17], even if households are fully aware of the mortal danger of rabies. Second, the level of children’s influence on this decision should be assessed before relying on children alone to filter important prevention information into households. Finally, given the results of the present study, it will be important to involve parents in households in both the above investigations, as they have been shown to be the actual decision-makers in terms of whether a household dog will be vaccinated, and whether a child with a dog bite will be taken for a course of preventative vaccine. Methodology which identifies and consults local decision-makers directly in this way can help incorporate more dynamic, multi-dimensional communication mechanisms to make interventions more effective and more sustainable.

Our findings rely on data from two different surveys, and while they both originate from the same study population, the KAP survey looks at fewer households than the overall survey. An important limitation is that the overall data on which the analysis draws were gathered as part of a broader project with a more general aim. This made it difficult to explore some potentially interesting questions which emerged from the analysis, particularly if the original data contained insufficient information to support meaningful conclusions. An example involved a lack of information on the impacts of the father’s decision on vaccination, e.g. if vaccination of dogs is approved, are all dogs vaccinated, and if vaccination is free, is the father more inclined to allow vaccination? Details on the gender and age of children bringing the dogs for vaccination would also enrich the findings from this study. Equally, more work is required on how a wide range of household, community and professional actors involved in rabies prevention interact to make decisions.

## Conclusion

Understanding practices around dog ownership and care, as well as family decisions about the health of most at-risk groups such as children, can help to direct rabies-prevention resources more directly to the people making these decisions. Specifically, the present study suggests a possible strategy for improving recommended (>70%) dog vaccination coverage in communities by identifying and consulting household members who make decisions on the issue. Veterinary officers and village elders should be approached to find the households owning dogs. It also suggests consulting household decision-makers about the dynamics involved in whether or not to seek PEP for children bitten by dogs. This more inclusive, yet targeted, approach is more likely to facilitate and maintain changes in practice in households than focusing more generally on awareness in at-risk groups alone. The results provide a basis for further research to identify the dynamics involved in this household decision-making, recognising that individuals may experience obstacles complying with prevention guidelines, even when they are aware of best practice.

## Supporting information

S1 Text A: Qualitative data collection _ Questions guide. B: Household questionnaire. C: Knowledge Attitudes and Practices questionnaire.
(DOCX)
