# Supplementary material for: Dog ownership practices and responsibilities for children’s health in terms of rabies control and prevention in rural communities in Tanzania
Source: PLoS Negl Trop Dis. 2021 Mar 10;15(3):e0009220. doi: 10.1371/journal.pntd.0009220 (PMC7946275; doi:10.1371/journal.pntd.0009220)
Supplement: S1 Text — A: Qualitative data collection _ Questions guide. B: Household questionnaire. C: Knowledge Attitudes and Practices questionnaire. (DOCX) [file pntd.0009220.s001.docx]

**Supporting information**

**Dog ownership practices and responsibilities for children’s health in terms of rabies control and prevention in rural communities in Tanzania**

**Lwitiko Sikana^1,2,3^, Tiziana Lembo^3^, Katie Hampson^1,3^, Kennedy Lushasi^1,2,3^ , Sally Mtenga^1^, Maganga Sambo^1,3^ , Daniel Wight^4^, Jane Coutts^3^ , Katharina Kreppel^2,3*^**

**1** Environmental Health and Ecological Sciences, Ifakara Health Institute, P.O. Box 53, Ifakara, Tanzania, **2** Global Health and Biomedical Sciences, School of Life Sciences and Bio-Engineering, Nelson Mandela African Institution of Science and Technology, P.O. Box 447, Arusha, Tanzania, **3** Boyd Orr Centre for Population and Ecosystem Health, Institute of Biodiversity, Animal Health and Comparative Medicine, University of Glasgow, Glasgow G12 8QQ, United Kingdom, **4** Medical Research Council, Social and Public Health Sciences Unit, University of Glasgow, Glasgow G2 3AX, United Kingdom.

***** katharina.kreppel@nm-aist.ac.tz

**A: Qualitative data collection _ Questions guide**

**Veterinary workers:**

- How many staff operates in their department, and to whom do they report at regional and national level?
- Where are their nearest laboratory services for sending samples? Do they ever send away samples and keep records about them?
- What are the main reasons for any contact they have with people in the target villages (e.g. livestock treatment, disease control, etc.)?
- Do they ever vaccinate dogs? If they do not, do they know whether people largely use private veterinary services if they vaccinate their dogs?
- Who typically brings the animal for vaccination?
- If they are children, which age-group do they typically belong to?
- Do they know how far the person has had to come?
- If the vaccination has been repeated, have the same dogs returned for regular vaccination?

**Health workers:**

- Is there a formal protocol at the health centre for treating bite victims?
- If someone comes in with a bite wound, who is the first person they see at the health centre? Does this person know anything about rabies and who would they refer the patient to?
- Does someone explain to bite victims that they will be given a course of four vaccinations and that they need to return on specific days?
- Does someone explain why this is important?
- If the bite victim is a child, who typically accompanies them to the health centre?
- If the bite victim is a child, who are the explanations given to, the child or the person accompanying them?
- Does anyone at the health centre record information about the bite and the victim? If so, what? Do they, for example, take any contact details for the person, or ascertain how far away they live?
- Stock of rabies vaccine, how it is acquired and how it is stored (cold chain information).
- Does the centre have access to supporting equipment, e.g. computer/printer; internet; mobile phones (company or personal)?
- Have they been involved in any public awareness campaigns on rabies?

**B. Household questionnaire**

Name of interviewer…………………………… Date………………………..

HOUSEHOLD ID ………………………

HOUSEHOLD DEMOGRAPHICS / DYNAMICS AND DOG CARE PATTERNS

- 1. How many people live in this household? [______]
  2. Dogs the household is responsible for:

| Name | Sex  M/F | Age mths | Age yrs |
| --- | --- | --- | --- |
|  |  |  |  |
|  |  |  |  |
|  |  |  |  |
|  |  |  |  |
|  |  |  |  |
|  |  |  |  |

- 1. Total n. of dogs <= 3 months [______]
  2. Total n. of dogs > 3 months [______]
  3. Complete the table below for each member of the household:

| Sex  M/F | Age  yrs | Marital status^1^ | Role in family^2^ | Education^3^ | Primary  occupation | Secondary occupation | Religion | Do you look after household dogs? | Do you take household dogs for vaccination? |
| --- | --- | --- | --- | --- | --- | --- | --- | --- | --- |
|  |  |  |  |  |  |  |  |  |  |
|  |  |  |  |  |  |  |  |  |  |
|  |  |  |  |  |  |  |  |  |  |
|  |  |  |  |  |  |  |  |  |  |
|  |  |  |  |  |  |  |  |  |  |

^1^ M married, S single, W widowed, D divorced

^2^ Household head, Relative to household head: spouse, son, daughter, brother, sister, father, mother, nephew, niece etc

^3^ Highest level of education reached

^4^ F employed on farm, S self-employed off farm, E employed off farm – agriculture, salaried, other

- 1. For each member of the household aged < 18 years, also complete the table below:

| School  Y/N | N. of hours spent in out-of-school activities | Type of out-of-school activities | Where do you spend most of the morning? | Where do you spend most of the afternoon? | Where do you spend most of the evening? | Where do you spend most of the night? |
| --- | --- | --- | --- | --- | --- | --- |
|  |  |  |  |  |  |  |

- 1. More detail on who mostly influences children and young people during the week:

| In the place where this person spends most of the morning, who is their main point of reference/influence? | In the place where this person spends most of the afternoon, who is their main point of reference/influence? | In the place where this person spends most of the evening, who is their main point of reference/influence? | In the place where this person spends most of the night, who is their main point of reference/influence? |
| --- | --- | --- | --- |
|  |  |  |  |
|  |  |  |  |
|  |  |  |  |
|  |  |  |  |

- 1. More detail on who mostly influences children and young people during the weekend:

| In the place where this person spends most of the morning, who is their main point of reference/influence? | In the place where this person spends most of the afternoon, who is their main point of reference/influence? | In the place where this person spends most of the evening, who is their main point of reference/influence? | In the place where this person spends most of the night, who is their main point of reference/influence? |
| --- | --- | --- | --- |
|  |  |  |  |
|  |  |  |  |
|  |  |  |  |
|  |  |  |  |
|  |  |  |  |

- 1. Who in the household decides whether:
- Dogs are vaccinated…………………………………….
- A child is taken for PEP if they are bitten……………………………………….
  1. If dogs are vaccinated, where are they taken for vaccination? ……………………………………….

ASSETS AND INCOME

*ASSETS*

- 1. Does any member of household own any of the following assets?

| Asset | Number of  Units | Purchase Price TSh  (If purchased) | Age | Working  Y/N |
| --- | --- | --- | --- | --- |
| Ox plough |  |  |  |  |
| Ox cart |  |  |  |  |
| Bicycle |  |  |  |  |
| Motorbike |  |  |  |  |
| Motor Vehicle |  |  |  |  |
| Tractor |  |  |  |  |
| Mobile phone |  |  |  |  |
| Radio |  |  |  |  |
| TV |  |  |  |  |

*HOUSE DETAILS*

- 1. Is your house rented? YES / NO
  2. How many houses are there? [______]
  3. How many other buildings are there? [______]
  4. Do any living quarters have a metal roof? YES / NO
  5. Are any living quarters built of concrete block or brick? YES / NO
  6. What is the number of rooms in all living quarters combined?............................
  7. Latrine? (circle)

1. indoor 2. outdoor 3. none

- 1. Electricity? 1. none 2. grid 3. off grid 4. if off grid, specify………………………………..
  2. What is your primary water source now? (circle respective number):

1. private well 2. community well 3. river 4. pond 5. other (mention): …….............………....................

- 1. How long (hours) does it take you to travel to obtain drinking water (one way)? ……………………………………………….
  2. How many times per week do you go to obtain drinking water?.......................................................................
  3. What are the energy sources you use for cooking (circle):

1. electricity 2. gas 3. Kerosene 4. cow dung 5. firewood 6. charcoal

7. Other (mention)…………………………………………

- 1. How long (hours) does it take you to travel to collect firewood (one way)? …………………………………………………….
  2. How many times per week do you go to collect firewood?.................................................................................

*LAND USE*

- 1. Is the land you use for grazing:

1. owned by you 2. rented from others 3. common land 4. Other (mention):......................

- 1. Is the land you plant for crops:

1. owned by you 2. rented from others 3. mcommon land 4. Other (mention):.......................

- 1. Is the land where the house is built:

1. owned by you 2. rented from others 3. common land 4. other (mention):........................

*INCOME SOURCES (Use ticks)*

i) Livestock sales ( )

ii) Milk sales ( )

iii) Other livestock income ( )

iv) Crops related ( )

v) Honey related ( )

vi) Wildlife related ( )

vii) Food relief ( )

viii) Off-farm employment ( )

*CROPS*

- 1. Have you harvested any crops in the past four months? YES / NO

If **YES**, fill in the table below for crops harvested:

|  | Crops harvested | | Crops sold | | | Crops given away to others | |
| --- | --- | --- | --- | --- | --- | --- | --- |
|  | No. (units) | Month | No.  (units) | Price per unit | Month sold | No. (units) | Month |
| Rice |  |  |  |  |  |  |  |
| Millet |  |  |  |  |  |  |  |
| Maize |  |  |  |  |  |  |  |
| Sesame |  |  |  |  |  |  |  |
| Cassava |  |  |  |  |  |  |  |
| Sweet potato |  |  |  |  |  |  |  |
| Bean |  |  |  |  |  |  |  |
| Cabbage |  |  |  |  |  |  |  |
| Lettuce |  |  |  |  |  |  |  |
| Vegetable |  |  |  |  |  |  |  |
| Tomato |  |  |  |  |  |  |  |
| Banana |  |  |  |  |  |  |  |
| Cotton |  |  |  |  |  |  |  |
| Other  …………………. |  |  |  |  |  |  |  |

- 1. LIVESTOCK IN THE HOUSEHOLD

| Type of animal | If Yes, how many |
| --- | --- |
| Cattle | \|___\|___\|___\|____\| |
| Goats | \|___\|___\|___\|____\| |
| Sheep | \|___\|___\|___\|____\| |
| Pigs | \|___\|___\|___\|____\| |
| Donkeys | \|___\|___\|___\|____\| |
| Chicken | \|___\|___\|___\|____\| |
| Others…………………. | \|___\|___\|___\|____\| |
| Others…………………. | \|___\|___\|___\|____\| |

RABIES / BITE INCIDENCE

- 1. Have you heard of any rabies incidents in animals in the past 2 yrs in the village? YES / NO

If YES, take the following details:

| Species | Date of case | Some description of incident if possible |
| --- | --- | --- |
|  |  |  |
|  |  |  |
|  |  |  |
|  |  |  |
|  |  |  |
|  |  |  |
|  |  |  |

Have you heard of any rabies incidents in humans in the past 5 yrs in the village? YES / NO

If YES, take the following details:

| Name | Age | Sex | Date of case | Clinical signs | Date of bite | Species  biting | Hospital Y / N | PEP Y / N | PEP completed Y / N | Died/  recovered | If died,  date |
| --- | --- | --- | --- | --- | --- | --- | --- | --- | --- | --- | --- |
|  |  |  |  |  |  |  |  |  |  |  |  |
|  |  |  |  |  |  |  |  |  |  |  |  |
|  |  |  |  |  |  |  |  |  |  |  |  |
|  |  |  |  |  |  |  |  |  |  |  |  |
|  |  |  |  |  |  |  |  |  |  |  |  |
|  |  |  |  |  |  |  |  |  |  |  |  |

- 1. Has any member of your family been exposed to a suspect rabid animal? YES / NO

If YES, take the following details:

| Age | Date bite | Species biting | Clinical signs of bite victim | Bite victim alive / dead | PEP Y / N | PEP completed Y / N |
| --- | --- | --- | --- | --- | --- | --- |
|  |  |  |  |  |  |  |
|  |  |  |  |  |  |  |
|  |  |  |  |  |  |  |
|  |  |  |  |  |  |  |

- 1. Do you know anyone else in the village who has been exposed to a suspect rabid animals?

YES / NO

If **YES**, take the following details:

| Age | Date bite | Species biting | Clinical signs of bite victim | Bite victim alive / dead |
| --- | --- | --- | --- | --- |
|  |  |  |  |  |
|  |  |  |  |  |
|  |  |  |  |  |
|  |  |  |  |  |

**C: Knowledge, attitude and practice (KAP) questionnaire**

Do not prompt for answers. If multiple choices are available, use codes (numbers) to record the answer. More than one answer is possible.

INTERVIEWER / INTERVIEWEE IDENTIFICATION

Name of interviewer…………………………… Date………………………..

*ID code of interviewee…………………………………………………*

RESPONSIBILITY FOR DOGS IN THE HOUSEHOLD

1. How many people live in your household? [_________]
2. N. of dogs <= 3 months the household is responsible for [_________]
3. N. of dogs > 3 months the household is responsible for [_________]
4. How important are dogs to you / your household? Rank the following species in order or importance (1=very important, 7=not very important)

| Type of animal | Do you own any? Y/N | Rank how important they are to you |
| --- | --- | --- |
| Cattle |  |  |
| Goats |  |  |
| Sheep |  |  |
| Pigs |  |  |
| Donkeys |  |  |
| Chicken |  |  |
| Dogs |  |  |
| Cats |  |  |
| Others…………………. |  |  |
| Others…………………. |  |  |

1. Why have you ranked them in this order?.......................................................................
2. Who in the household is responsible for dogs vaccination?………………………………………………
3. Do you think that vaccinating dogs against rabies is important? YES / NO
4. If YES, why?.......................................................................................................................
5. If NO, why not? .......................................................................................................................
6. Who would take them for rabies vaccination if this was provided?....................................................
7. Where would you take them for rabies vaccination if this was provided?.......................................
8. How many dogs < 3 months vaccinated against rabies in previous year? [___]
9. And how many > 3 months? [___]
10. If dog/s vaccinated, can you show me the vaccination certificate/s?

YES (tick if seen certificate)

YES but uncertain where it is stored

NO

1. If dog/s not vaccinated, what was the reason?

1= no vaccination carried out in the area

2= costs

3= it will stop dogs fulfilling their function in the household

4= other, specify…………………………………………………………………………

KNOWLEDGE ABOUT RABIES AND RABIES PREVENTION

1. Have you ever heard of rabies? YES / NO
2. If YES, where did you get knowledge of rabies for the first time?

1=because I have seen it personally

2= from TV

3= from radio

4= from newspaper [___] [___] [___]

5= from school

6= from government /community meeting

7= from poster/leaflets/ brochure

8= from local community (parent/neighbour/friend etc)

9= other, specify………………………………..

1. If YES, can you describe what rabies looks like? ....................................................................................…………………………………………………………................................………………………………....………....................................................................................................................................................................
2. How can rabies be caught? .......................................................

1= through a bite,

2= through a scratch [___]

3= do not know

4= other

Which species of animal can transmit rabies?

1= humans

2= snakes

3= chickens

4= dogs

5= cats [___] [___] [___]

6= cattle

7= hyenas

8= goats

9= mongoose

10=another way? Specify……………………

1. What would you do if you saw a dog behaving strangely or aggressively?

1= throw something at it

2= make a noise to scare it away

3= try to separate it from other dogs

4= report it to someone in authority and make sure no one went near it

1. What is the first thing you should do if you or any family member is exposed to any suspect animal bites?

1= wash wound with soap and water

2= report to police

3= wash with kerosene [___]

4= go to see healer

5= go to hospital

6= other specify……………………………………

1. What is the second thing you should do if you or any family member is exposed to any suspect animal bites?

1= wash wound with soap and water

2= report to police

3= wash with kerosene [___]

4= go to see healer

5= go to hospital

6= other specify……………………………………

1. If respondent says wash wound with soap and water, how quickly should you do this? ………………………............................................................................................................................................................................................................................................................
2. If respondent says wash wound with soap and water, where have you learnt this?

1= from TV

2= from radio

3= from newspaper [___] [___] [___]

4= from school

5= from government /community meeting

6= from poster/leaflets/ brochure

7= from local community (parent/neighbour/friend etc)

8= other, specify………………………………..

1. If respondent says **go to hospital**, how quickly should you do this? ………………………...
2. If respondent says **go to hospital,** where have you learnt this?...………………………...

1= from TV

2= from radio

3= from newspaper [___] [___] [___]

4= from school

5= from government /community meeting

6= from poster/leaflets/ brochure

7= from local community (parent/neighbour/friend etc)

8= other, specify………………………………..

1. If you were to go to hospital, what treatment would you expect at the hospital?

1= antibiotics

2= painkiller

3= vaccine (anti-rabies) [___] [___] [___]

4= tetanus

5= dress wound

6= would take advice of the staff

7= other, specify……………………………………

1. If you were NOT to go to hospital, why?

1= it is too far away

2= it is too expensive

3= it is only a bite and it will heal [___] [___] [___]

4= I was told that it is not necessary

5= other, specify……………………………………

1. If the answer is 4 (I was told that it is not necessary), who told you this?.......................................
2. How would you get to the hospital?

1= on foot

2= by bike

3= by car [___] [___] [___]

4= by public transport

5= other, specify……………………………………

1. If it was a child that was bitten and needed to be taken to the hospital, who would take him / her?...............................................................................................................................................................................
2. Would the person taking a child to the hospital lose income in the time they are away?..................................................................................................................................................................
3. What use do you make of the health system more generally?......................................................
4. Do you regularly seek health care for anything else? YES / NO.

If **YES**, provide examples………………………………………………………………………………………………………………..

1. What would you do to a suspect rabid animal that attempts to bite you/your family member/neighbour)?

1= immediately kill that animal

2= report to livestock office [___] [___] [___]

3= I would not take any action

4= other, specify……………………………………

1. If the answer is 1 (to kill the animal) what action would you take with the killed animal?

1= throw away

2= burn [___] [___] [___]

3= bury

4= cut head and send to livestock office

1. Which would you fear most, (1) having malaria or (2) being bitten by a rabid animal?
2. Why: …………………………………………………………………………………………
3. Is there any cure/treatment for a person who started developing rabies symptoms? YES / NO
4. If YES mention it………………………….……………………….…………………………
5. Do you know ways to control rabies in animals? YES / NO
6. If YES mention them:

i……………………………………………………………….

ii……………………………………………………………….

iii…………………………………………………..………….

iv.……………………………………………………………..
